# ARN: Analysis and Visualization System for Adipogenic Regulation Network Information

**DOI:** 10.1038/srep39347

**Published:** 2016-12-16

**Authors:** Yan Huang, Li Wang, Lin-sen Zan

**Affiliations:** 1 National Beef Cattle Improvement Center, College of Animal Science and Technology, Northwest A&F University, Yangling, China

## Abstract

Adipogenesis is the process of cell differentiation through which preadipocytes become adipocytes. Lots of research is currently ongoing to identify genes, including their gene products and microRNAs, that correlate with fat cell development. However, information fragmentation hampers the identification of key regulatory genes and pathways. Here, we present a database of literature-curated adipogenesis-related regulatory interactions, designated the Adipogenesis Regulation Network (ARN, http://210.27.80.93/arn/), which currently contains 3101 nodes (genes and microRNAs), 1863 regulatory interactions, and 33,969 expression records associated with adipogenesis, based on 1619 papers. A sentence-based text-mining approach was employed for efficient manual curation of regulatory interactions from approximately 37,000 PubMed abstracts. Additionally, we further determined 13,103 possible node relationships by searching miRGate, BioGRID, PAZAR and TRRUST. ARN also has several useful features: i) regulatory map information; ii) tests to examine the impact of a query node on adipogenesis; iii) tests for the interactions and modes of a query node; iv) prediction of interactions of a query node; and v) analysis of experimental data or the construction of hypotheses related to adipogenesis. In summary, ARN can store, retrieve and analyze adipogenesis-related information as well as support ongoing adipogenesis research and contribute to the discovery of key regulatory genes and pathways.

Obesity, the excess deposition of adipose tissue, is among the most pressing health problems both in the Western world and in developing countries. Growth of adipose tissue is mainly the result of the development of new fat cells from precursor cells. This process of fat cell development, known as adipogenesis, leads to the accumulation of lipids and an increase in the number and size of fat cells^1^. At the cellular level, a large number of transcription factors^2^, epigenetic modification factors^3^, microRNAs^4^, signal factors^5^ and splicing factors^6^ are involved in the regulation of adipocyte differentiation, and the regulatory interactions between all of these factors constitute a complex regulatory network.

Existing databases are focused on certain types of regulatory factors and interactions, including PAZAR^7^ and TRRUST^8^ for transcription factors, miRGate^9^ for microRNAs, PIPs^10^ and BioGRID^11^ for protein–protein interactions and EpiFactors^12^ for epigenetic factors. However, there is no cross-referencing between the results of experimental studies on adipogenesis and these databases due to the lack of an adipogenesis information center. Moreover, new studies on adipogenesis are continuously published and complicate the acquisition of information for specific research purposes and questions.

The Adipogenesis Regulation Network (ARN) is a database of molecule-molecule regulatory interactions identified via the manual curation of PubMed abstracts. For efficient curation of a large number of PubMed abstracts, we used a sentence-based text-mining approach in which text sentences that might pertain to adipogenesis regulation were first extracted and then subjected to manual curation. The current version of ARN contains 53,655 records related to adipogenic differentiation, which to our knowledge constitutes the largest public database of literature-curated adipogenesis regulatory interactions to date. Moreover, the majority of the interactions have annotations for mode-of-regulation (i.e., activation or repression), and most of the nodes have annotations for classification, impact and function. In addition, by incorporating records of external databases, ARN provides 13,103 predictive relationships that may be related to adipocyte differentiation. Importantly, ARN provides an online tool with filtering and analysis functions, suggesting that ARN will be a useful benchmark for the development of hypotheses regarding adipogenesis.

## Results

### Database Description

**Figure 1:**
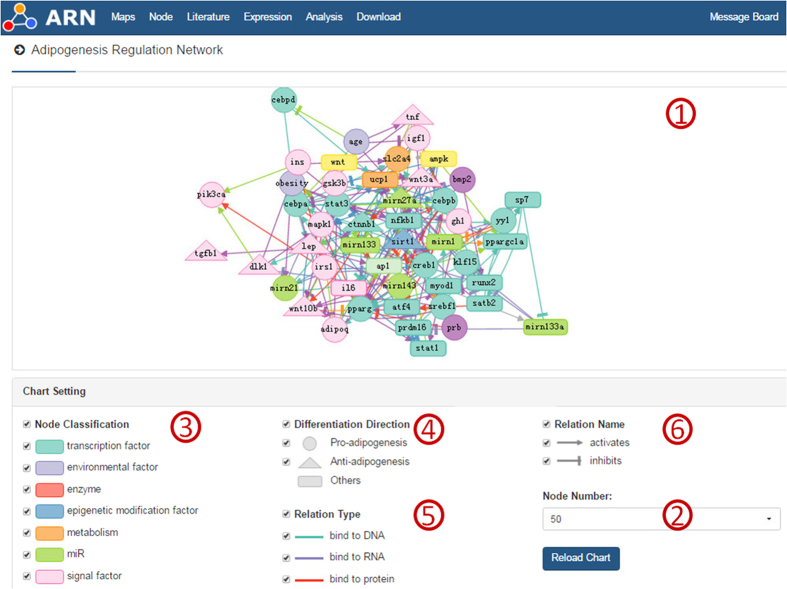
Visualization of 50 hub nodes in adipogenesis. This database screenshot shows the main home page. ① Visualization network of the top 50 highly connected nodes. ② Here, the user chooses the number of the nodes that he or she wants to see. ③ The color of the node is determined by its classification. ④ The shape of the node is determined by its function during the process of adipogenesis. ⑤ The relationship type determines the color of the links. ⑥ The relationship name determines the shape of the link. (If the user selects the check box in the map, the display content can be customized).

The homepage of the database provides a visualization of the adipogenesis regulation network, which consists of 50 nodes with the largest numbers of connections. Users can choose the number of nodes they wish to view. The color and shape of a node is determined by its classification and function, and the color and shape of a link is determined by its impact and mode of action (Fig. 1).

**Figure 2:**
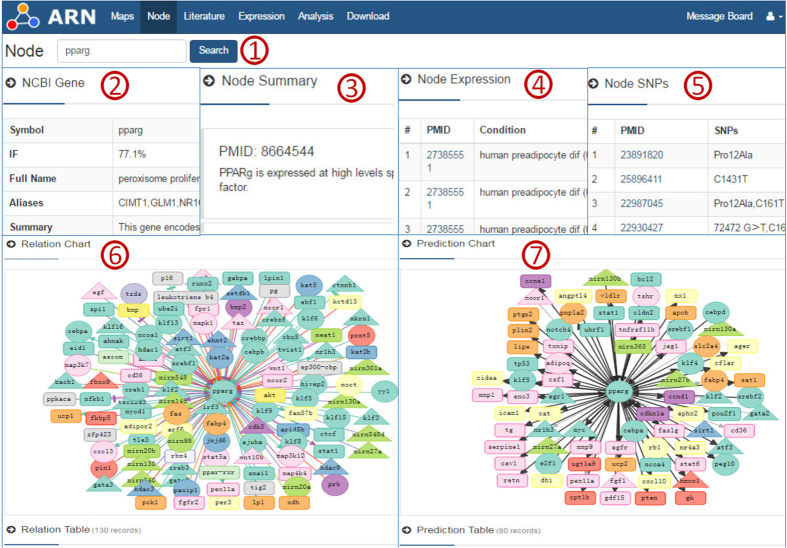
Screenshot of the node page of the ARN database. These database screenshots show the main results page for a gene search and the corresponding relationships network using the example of PPARg. ① The search window enables the user to search for a gene or preform a microRNA query. ② IF and Link Number describe the effects of the gene on adipogenesis. ③, ④ and ⑤ Table providing the Summary, SNPs, Expression and related papers for the gene. ⑥ Visualization of the relationship network. The contents of the chart correspond to those in the table. ⑦ Visualization of the prediction network. The contents of the chart correspond to those in the table. This figure does not display all results, although full information is on the website.

The node page consists of six sections (Fig. 2). The first section lists general information for the requested gene or microRNA. The second section contains a list of sentences describing the gene or microRNA in the context of adipogenesis and the corresponding PMID. The third section contains a table that shows the expression of the node under different conditions. The fourth section contains a table showing SNPs associated with the node. The fifth section consists of a relationship chart and a visualization network, and the color and shape of the node and the link are identical to those of the homepage. The final section is a relationship table that can be filtered according to potential impact (e.g., activation or inhibition), mode of action (e.g., DNA binding or epigenetic modification) and test method (e.g., ChiP or siRNA). Users can also order the results by the impact factor (IF) of the source and target nodes. Moreover, possible relationships (TFs and miR targets) associated with the node are accessible in the sixth section of the page, which contains a visualization network in addition to the table. New predictions are shown with bold black links.

On the maps page (Fig. 3①), we provide images collected from review papers on adipogenesis. These images were divided into six categories: epigenetic modification, transcription regulation, signal transduction, miR, cell growth and others. Below every picture, a table lists all of the nodes in the picture. By clicking on a gene symbol, the user is directed to the node page for the specific gene. We also provide a network of these nodes based on our database.

The literature page (Fig. 3②) provides basic information about the articles. All papers were divided into four categories (review, article, SNPs and high-throughput) according to their contents and results. We then manually extracted the materials and methods used in each paper.

Moreover, the expression of genes involved in adipogenic differentiation progression is available on the expression page (Fig. 3③). Users can view a line chart by clicking on the button following it. Expression data were collected from many different papers. Comparisons of these data facilitate access to different perspectives to understand gene functions.

We also provide a download page (Fig. 3③). Users can choose one class of genes (e.g., “transcription factors” under “Classification” and “promoters of adipogenesis” under “Differentiation Direction”) and then download the GeneIDs, symbols and PMIDs for related papers. This information can also be directly used to search other databases.

If we have missed specific genes or publications regarding adipogenesis, users are welcome to send suggestions via the ARN message board, and we are pleased to add them to the database. A graphical guide of the ARN database is available for download on the database website at http://210.27.80.93/arn/.

### Application of the ARN database

#### Basic Search

The database can be searched online (http://210.27.80.93/arn/) with three possible input forms depending on the user’s research focus (See Supplementary materials, ARN Handbook, Example 1). For gene searches, Entrez GeneID and official gene symbols are accepted. MicroRNAs require names of mature microRNA sequences (e.g., mirn143). Literature searching requires a PubMed PMID. Users can select their requested entry, and the results page is displayed. In practice, the most important contents are the four following types of information. i) Regulatory map information. The ARN Map page provides graphics summarized by experts in the field of adipogenesis. ii) Impact of a query node on adipogenesis. The “IF” value measures the degree of influence, while “differentiation direction” represents the nature of the impact; for example, circular nodes indicate that the node promotes adipogenesis, whereas triangular nodes indicate that the node inhibits fat formation. iii) Interactions and their mode for a query node. In the relationship chart for an ARN Node page, the shapes and colors of the links represent information on interactions and their modes. iv) Prediction of interactions of a query node. The Prediction Chart contains the predicted relationships for a query node based on four external databases (miRGate, BioGRID, PAZAR and TRRUST); bold links show prediction relationships that are new, whereas gray links indicate that these prediction relationships have been verified to be involved in the regulation of adipogenesis.

When we searched “PPARg” in NCBI PubMed, we obtained more than 900 results. Users may then read through the list of results one by one. When we searched “PPARg” in the ARN database, the results page included six sections, as shown in Fig. 2 and Table 1, with data collected from seven websites (NCBI-Gene, miRBase, NCBI-PubMed, miRGate, PAZAR, TRRUST, and BioGRID) and 109 papers. Among the sections on the results page, “NCBI gene” and “Literature summary” provide an overall summary of the PPARg gene as well as a summary from a professional point of view, respectively; “Node Expression” and “Relation Chart” show information on what is known about PPARg; and finally, “Prediction Chart” lets us identify potentially new studies on the regulation of adipogenesis. Furthermore, users can sort based on the “IF” value of the nodes in the “Prediction Table” to select the most important predictions. Table 2 provides examples of prediction results. For example, the results indicate that Pan *et al*.^13^ demonstrated that both E2F1 and CEBPd are involved in the transcriptional regulation of PPARg in cancer cells in the process of apoptosis. Thus, researchers can design experiments to verify the effects of E2F1 and CEBPd on adipogenic differentiation by PPARg.

#### Analysis of experimental data and construction of hypotheses

**Figure 4:**
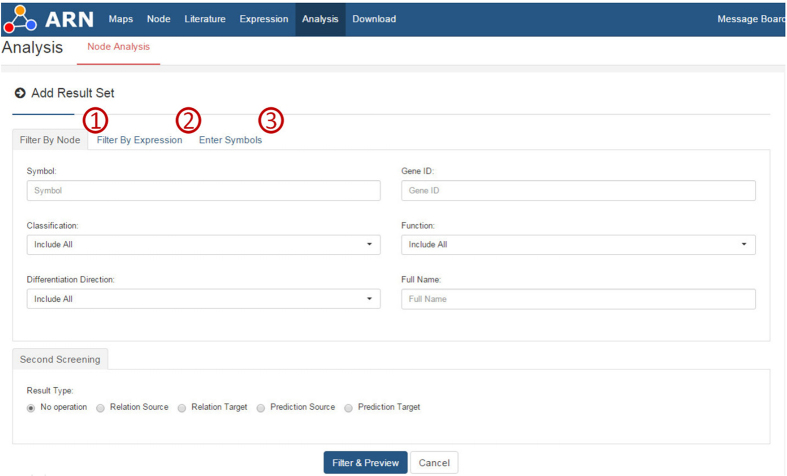
ARN Analysis tool. The Analysis tool can perform a two-step analysis for 3 types of data. The first step is the selection of specific node sets. The second step is the analysis of the intersection of node sets. ①, ②, and ③ indicate the three types of data used for analysis.

**Figure 5:**
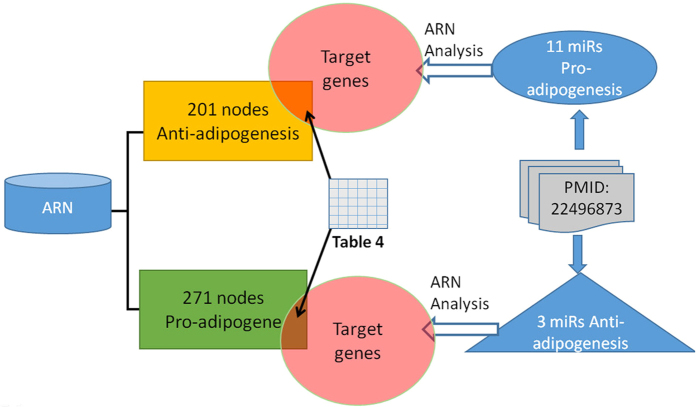
Analysis of PMID-22496873.

Currently, the database contains over 53,000 records. Such a large amount of information represents a solid foundation for analysis and prediction. The ARN database provides 2 useful analytical tools for the user: (1) the “IF” value of each node allows us to gauge the extent of the impact of the node on adipogenesis, whereas the (2) ARN Analysis page allows users to perform analyses based on a node or a class of nodes, an article or a specific node set in the ARN Analysis page (see Fig. 4). For example, Chartoumpekis *et al*.^14^ (PMID: 22496873) analyzed the miRNA expression profile of adipose tissue after long-term high-fat diet-induced obesity in mice using microarray analysis and identified 25 differentially expressed microRNAs. First, we need to rapidly screen miRNAs to identify those that are highly correlated with adipogenesis. The ‘IF’ value is very useful in this case, as a greater ‘IF’ of a node corresponds to a greater effect on adipogenesis. Table 3 shows detailed information. Four out of 10 up-regulated microRNAs have been confirmed to promote or inhibit adipogenesis, whereas 10 out of 15 down-regulated microRNAs have been confirmed to promote or inhibit adipogenesis. Once we have identified the object of study, the ‘ARN Analysis’ page is useful. Thus, we need to identify the intersection between their target genes and pro-osteogenesis genes or the intersection between their target genes and anti-adipogenesis genes (see Fig. 5). ‘ARN Analysis’ is helpful for identifying these intersections, and we can obtain the results shown in Table 4 (Analysis steps: see Supplementary materials “ARN Handbook” - Example 4).

## Discussion

There is ongoing research to detect genes or pathways that are frequently altered in adipogenesis. Identification of such genes and pathways becomes more complicated due to the ever increasing body of literature containing adipogenesis studies, making literature searches highly time-consuming. Therefore, it is necessary to structure the existing knowledge of genes and microRNAs associated with adipogenesis. To this end, we developed the ARN database to provide a review of the current state of adipogenesis research, and we have made this information easily accessible to researchers.

### Hub nodes in adipogenesis

The ultimate aim of adipogenesis research is to understand the molecular mechanisms underlying the biology of obesity to discover innovative prognostic and/or predictive biomarkers. Table 5 lists the top 50 genes or microRNAs and the corresponding number of relationship records. This table is ranked according to the possible impact of the genes or microRNAs.

Until now, prognostic predictions or therapeutic stratification of obesity have not been based on biomarkers. However, the table suggests many promising candidates that should be further investigated, potentially in clinical studies.

### Target control of adipogenesis genes

Target control refers to the control of a subset of target nodes (or a subsystem) that are essential for a system’s mission pertaining to a selected task^15^. If we know all the relationships for a given node, then we may understand how to control it. The ARN database provides an overall view of each node in the adipogenesis regulation network. As shown in Fig. 2 for the node PPARg, there is a map comprising the full life cycle of this protein, from epigenetic modification of its chromatin^16^,^17^,^18^,^19^,^20^, transcriptional regulation of its promoters^21^,^22^,^23^,^24^,^25^,^26^,^27^, post-transcriptional regulation by microRNAs^28^,^29^,^30^,^31^, phosphorylation of its proteins by signal factors^32^,^33^, transcription initiation to final degradation. Such detailed knowledge of PPARg may help us design an ideal path for its control.

### Future directions

Mesenchymal stem cells (MSCs), the precursors of adipocytes, can also differentiate into osteoblasts, chondrocytes and myoblasts. Understanding the factors that govern MSC differentiation has significant implications in diverse areas of human health, from obesity to osteoporosis to regenerative medicine^34^. Thus, we would like to add these MSC differentiation factors into our network in the future. Moreover, it was recently shown that long-chain non-coding RNA (lncRNA) is involved in the regulation of adipogenic differentiation^35^,^36^; thus, lncRNA data must be added as soon as they are available. In addition, information regarding the institutions involved in the papers included in the database will soon be available for visualization, and we expect that this will promote the exchange of ideas, project cooperation and resource sharing between institutions. We plan to update the database monthly to provide state-of-the-art knowledge and track improvements in the field. All recently added data will be displayed separately on a corresponding page.

We hope that the ARN database will serve as a platform for information and hypothesis generation for the research community and will aid in elucidating the complexity of adipogenesis-related mechanisms, pathways and processes.

## Methods

**Figure 6:**
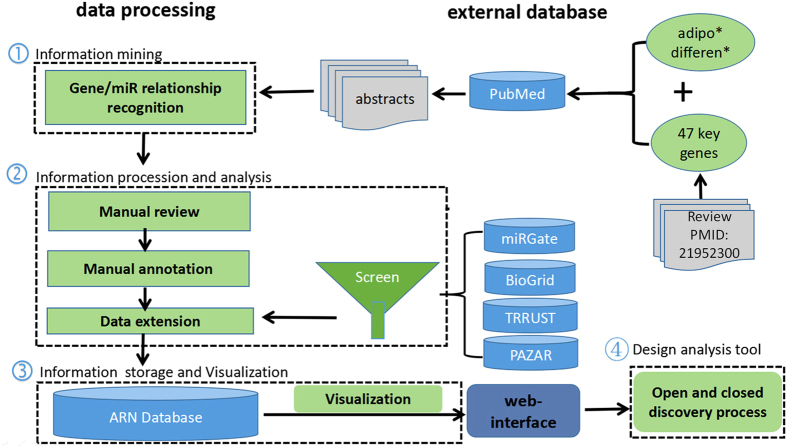
Database construction pipeline. This process is only used to build the initial network. When updating the data, only the keyword “adipocyte differentiation” was used for searching and analysis.

The ARN database aims to provide a high-quality collection of genes, microRNAs and relationships implicated in the regulation of adipogenesis, as reviewed by experts in the field. The data collection and processing steps are illustrated in Fig. 6. The workflow comprised four major steps as follows. Step one: construction of a text-mining association network using the Agilent Literature Search plugin^37^. Step two: manual review, annotation and extension. Step three: information storage and visualization. Step four: design of the analysis tool.

### Information mining

For the literature search, we built a set of queries by entering one of the key gene sets for adipogenesis^5^ and using the context ‘adipo* differen*’ (short for ‘adipocyte differentiation’). The query set was submitted to PubMed via Agilent Literature Search. The resulting documents were retrieved, parsed into sentences, and analyzed for known interaction terms, such as ‘binding’ or ‘activate’. Agilent Literature Search uses a lexicon set for defining gene names (concepts) and aliases, drawn from Entrez Gene, and interaction terms (verbs) of interest. An association was extracted for every sentence containing at least two concepts and one verb. Associations were then converted into interactions with corresponding sentences and source hyperlinks and added to a Cytoscape network. To choose key gene sets, we conducted a two-step procedure. In the first step, we established 47 key genes via a literature review^5^. This candidate set was updated by incoming nodes from post-manual curation. In the second step, we prioritized the remaining “candidate nodes” by scoring them based on the frequency of each node in all regulatory interactions; 53 new “candidate nodes” were used to search for candidate sentences for the next round of manual curation. The final download of abstracts was executed on 29 October 2015. In total, 9908 PubMed abstracts were obtained and served as the initial corpus for further processing. False positives for the results would not affect the quality of our database because molecular-molecular interactions would be identified by manual curation.

### Information processing and analysis

**Figure 7:**
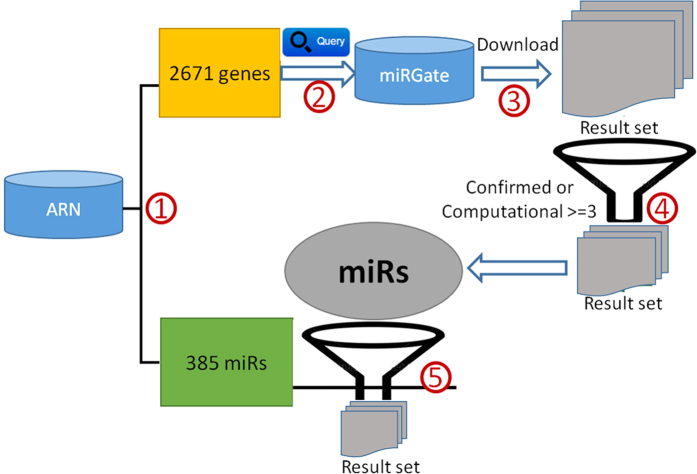
Screening the data from miRGate. Step ①: Screen all of the genes and miRs in the ARN database. Step ②: Submit these genes to the miRGate database. Step ③: Download the set of results. Step ④: Screen confirmed or computational predictions ≥3. Step ⑤: Screen the predictions with miRs in the ARN database.

During the manual review, annotation and extension step, the reviewers verified the specific genes, microRNAs and their relationships recognized in the abstracts. Additionally, information regarding experimental settings, node classification, function and adipogenic impact was marked. For each paper in the ARN database, the experimental settings comprised the experimental procedure, the names of cell lines and types of samples. Occasionally, a dormant value could only be revealed by combining one dataset with another, potentially a very different dataset. We screened data from 4 external databases and obtained more than 10000 prediction results from among over 1 million interaction records (Table 6). Using “miRGate” as an example, the screening process was as follows (Fig. 7). The workflow comprised five major steps as follows. Step one: We obtained 385 miRNAs and 2671 associated genes in the ARN database. Step two: We then submitted the 2671 genes to the miRGate database website (http://mirgate.bioinfo.cnio.es/miRGate/) for retrieval. Step three: The predicted results were downloaded. Step four: To obtain high-efficacy targets, we excluded target predictions with computational predictions of <3^9^. Step five: We used the 385 miRNAs recorded in the ARN database to screen the predictions. Finally, we obtained 8171 miRNA-Target prediction records, and after manual data cleaning, these were uploaded to the ARN database. The other three databases also underwent similar screenings. In the future, when a new database appears, we will be able to add data associated with adipogenic differentiation to the ARN database within a short period of time utilizing this method.

### Information storage and visualization

To store and access the collected information regarding the adipogenesis regulatory network, we implemented a database and a user-friendly web interface. The ARN database is a Microsoft SQL Server relational database. The table structure of the database is illustrated in Fig. S2; the complete content of this database is presented in Supplementary Table S1. To easily access the ARN, users can search and browse via a web interface at http://210.27.80.93/arn/. This interface was built on.NET and HTML5. For interactive data visualization, we applied D3 (d3js.org).

### Design analysis tool

**Figure 8:**
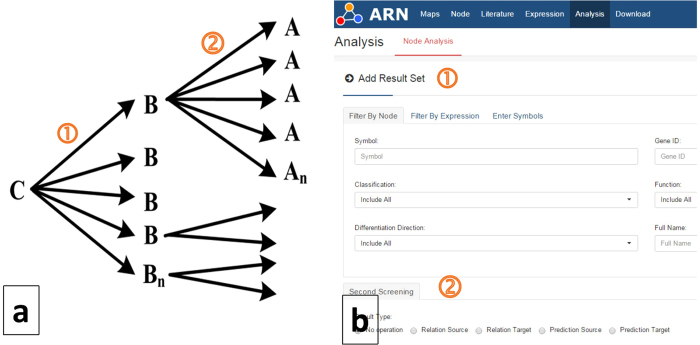
Open discovery process. (**a**) Open discovery process as defined by Weeber *et al*.^38^ (**b**) ARN Analysis open discovery tool.

Based on Swanson’s discovery process, Weeber *et al*.^38^ defined two types of knowledge discovery approaches: open discovery and closed discovery. An open discovery process is used to generate a hypothesis (Fig. 8a). For a given starting concept C, concepts that co-occur with C in the literature (called linking concepts B) are found. Concepts that co-occur with linking concepts B (called target concepts A) are then similarly found, bearing in mind that concepts A should not co-occur with starting concept C. This process can be described as C −> B −> A.

**Figure 9:**
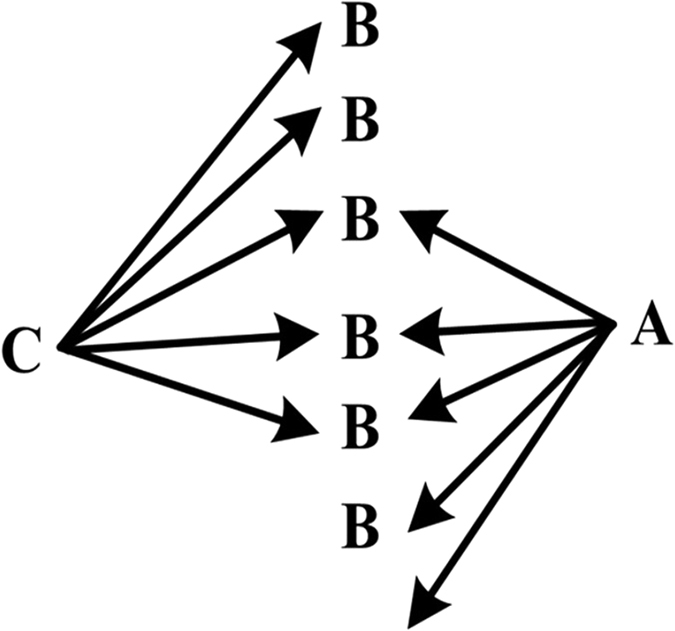
Closed discovery process as defined by Weeber *et al*.^38^. The process is a two-way discovery process starting from A and C simultaneously, followed by the discovery of intersection B.

A closed discovery process is used to test a hypothesis (Fig. 9). For two given concepts C and A, a researcher would like to determine whether or not hidden links exist between them. As more links are found between A and C, it is more likely that the tested hypothesis is correct. This process can be described as C −> B < −A.

We adopted the open discovery process to design a two-step discovery approach (Fig. 8b). Here, concept C is adipogenesis. Step 1 can screen out the nodes (called linking concepts B) that have specific effects on C. In Step 2, the second round of screening can identify concepts that co-occur with linking concepts B (called target concepts A).

We adopted the closed discovery process to design a discovery approach to identify two or more result sets. As shown in Fig. 5, we can obtain multiple result sets via the open discovery approach, and their intersections can be identified by ARN Analysis.

In the field of literature-based hidden knowledge discovery, popular methods based on co-occurrence produce too many target concepts, leading to the decreased ranking of potentially relevant target concepts. In this current paper, we propose a new method for choosing useful and promising linking concepts. This method calculated the “IF” value for each node according to the following formula:


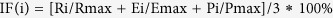


In this formula, IF (i) shows the effect of node i on the differentiation of fat. Ri indicates the number of relationships of node i, and Rmax indicates the number of relationships of node r-max, which has the greatest number of relationships; Ei indicates the number of expression records of node i. Emax indicates the number of expression records of node e-max, which has the greatest number of expression records; Pi indicates the number of prediction records of node i. Pmax indicates the number of prediction records of node p-max, which has the greatest number of prediction records. All values have been updated within the database, meaning that the information it contains is comprehensive and timely.

## Additional Information

**How to cite this article:** Huang, Y. *et al*. ARN: Analysis and Visualization System for Adipogenic Regulation Network Information. *Sci. Rep.* **6**, 39347; doi: 10.1038/srep39347 (2016).

**Publisher’s note:** Springer Nature remains neutral with regard to jurisdictional claims in published maps and institutional affiliations.

## Supplementary Material

Supplementary Dataset 1

Supplementary Material

## Figures and Tables

**Figure 3:**
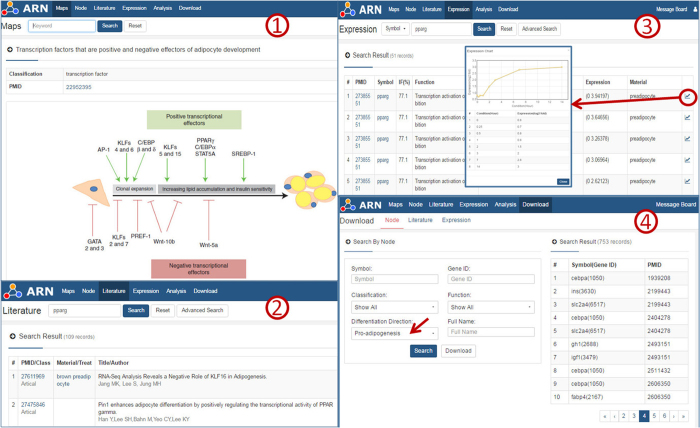
Screenshot of the other pages of the ARN database. The database screenshot shows the main results page for a gene search using the example of PPARg. ① The maps page shows images collected from review articles. A table lists all of the nodes in the image, and users can link to the node page for a specific gene by clicking on the gene’s name. ② The literature page provides all papers that reference the specific gene. ③ The expression page lists the expression of the specific gene during the process of adipogenesis under different conditions. Users can view a line chart by clicking on the button following it. ④ On the download page, users can choose one type of gene (e.g., transcription factors that promote adipogenesis) and then download related GeneIDs and PMIDs of related papers.

**Table 1:** The number of records in each ARN Node section for PPARg.

NO	Name	Number of records
1	NCBI Gene	7
2	Literature Summary	23
3	Node Expression	51
4	Node SNPs	6
5	Relation Chart and Table	130
6	Prediction Chart and Table	80
**Total**		297

**Table 2:** Top 10 PPARg prediction results.

NO	Source	IF (%)	Target	Agreement	Species	PMID
1	srebf1	27.7	pparg	100	Human	10409739
2	e2f1	22.1	pparg	100	Human	20971808
3	egr1	21.2	pparg	100	Human	12011097
4	cebpd	18.5	pparg	100	Human	20971808
5	mirn27a	17.0	pparg	100	Mouse	20060380
6	stat1	16.4	pparg	100	Human	15339920
7	sirt1	16.3	pparg	100	Human	15850715
8	mirn130b	14.2	pparg	100	Human	21135128
9	klf2	11.7	pparg	100	Human	12426306
10	mirn27b	10.4	Pparg	100	Mouse	22120719

The data in this table represent only part of the prediction results; the entire dataset can be viewed in the ARN database. The agreement is 100, which indicates a confirmed prediction.

**Table 3:** Twenty five differentially expressed microRNAs obtained by Chartoumpekis DV.

No	Up or down	Symbol	Direction of differentiation	IF (%)
1	Up-regulated	mirn335		19.1
2		**mirn21**	Pro-adipogenesis	16.7
3		mirn221		15.9
4		**mirn146b**	Pro-adipogenesis	14.6
5		mirn222		12.6
6		**mirn146a**	Pro-adipogenesis	11.7
**7**		**mirn142**	Anti-adipogenesis	9.3
8		mirn342		7.8
9		mirn379		4
10		mirn674		1.7
11	Down-regulated	**mirn30a**	Pro-adipogenesis	17.6
12		**mirn30e**	Pro-adipogenesis	16.7
13		mirn1		14.8
14		**mirn200a**	Pro-adipogenesis	9.8
15		mirn122		9.6
16		**mirn204**	Pro-adipogenesis	9
17		**mirn130a**	Anti-adipogenesis	8.4
18		**mirn141**	Pro-adipogenesis	8
19		**mirn192**	Anti-adipogenesis	7.8
20		**mirn200c**	Pro-adipogenesis	7.7
21		**mirn378**	Pro-adipogenesis	5.6
22		**mirn200b**	Pro-adipogenesis	5.6
23		mirn133b		5.6
24		mirn203		5.5
25		mirn193		3.9

Bold display microRNAs were selected for further analysis.

**Table 4:** Analysis results for PMID 22496873.

No	Direction of differentiation	Source	Category	Target	IF (%)
1	Pro-adipogenesis	mirn21	Prediction target	bach1	7.8
				nfat5	5.2
				reck	2.7
2		mirn204	Relation target	sirt1	16.3
				andcr	0.3
3		mirn146b	Relation target	brca1	29.8
				sirt1	16.3
			Prediction target	klf7	2
4		mirn200a	Prediction target	sirt1	16.3
				ctnnb1	20.7
				asxl1	6.9
				hdac4	9.2
				yap1	3,7
5		mirn146a	Relation target	kdm6b	4.7
				smad3	13.4
				wnt1	1.9
			Prediction target	brca1	29.8
6		mirn30a	Prediction target	tsc22d3	13.6
**7**		mirn30e	Prediction target	tsc22d3	13.6
				bach1	7.8
				nfat5	5.2
8		mirn200c	Prediction target	lepr	8
9		mirn378	Relation target	med13	0.9
10		mirn200b		None	
11	Anti-adipogenesis	mirn130a	Relation target	pparg	77.1
			Prediction target	ldlr	12.1
				ptger3	4.6
				mecom	1.2
12		mirn192	Relation target	scd	25.7
			Prediction target	rb1	15.2
				fndc3b	4
				ppp1cb	3.3
13		mirn142		None	

**Table 5:** Top 50 nodes in ARN.

No	Symbol	Links	No.	Symbol	Links
1	pparg	130	26	prdm16	18
2	cebpb	77	27	mirn1	18
3	runx2	65	28	cebpd	17
4	cebpa	52	29	tgfb1	17
5	ctnnb1	41	30	ap1	17
6	stat3	36	31	mirn133	16
7	creb1	34	32	satb2	16
8	srebf1	31	33	mirn143	16
9	nfkb1	30	34	wnt10b	16
10	mapk1	29	35	myod1	16
11	tnf	29	36	gsk3b	15
12	wnt*	28	37	mirn133a	15
13	sirt1	28	38	irs1	15
14	obesity*	26	39	pik3ca	15
15	mirn27a	25	40	prb	14
16	igf1	23	41	lep	14
17	adipoq	21	42	yy1	14
18	ampk	20	43	ins	14
19	ucp1	20	44	atf4	14
20	age*	20	45	wnt3a	13
21	ppargc1a	19	46	il6	13
22	bmp2	19	47	mirn21	13
23	klf15	19	48	dlk1	13
24	sp7	18	49	gh1	13
25	slc2a4	18	50	klf4	13

*Indicates that this node is not a gene or microRNA.

**Table 6:** Four external databases.

No.	URL	PMID	Relationship type	Total records	Records in ARN
1	http://www.grnpedia.org/trrust/	26066708	TFs-Targets	8215	3558
2	http://www.pazar.info/	18971253	TFs-Targets	6869	1080
3	http://mirgate.bioinfo.cnio.es	25858286	miRNAs-Targets	38810	8171
4	http://thebiogrid.org/download.php	16381927	Protein-Protein	1066335	282
