# Supplementary material for: ARN: Analysis and Visualization System for Adipogenic Regulation Network Information
Source: Sci Rep. 2016 Dec 16;6:39347. doi: 10.1038/srep39347 (PMC5159821; doi:10.1038/srep39347)
Supplement: Supplementary Material [file srep39347-s2.pdf]

ARN: Analysis and Visualization System for Adipogenic Regulation Network Information

Yan Huang[1]. Li Wang[1]. Lin-sen Zan[a1]

[1] National Beef Cattle Improvement Center, College of Animal Science and Technology, Northwest A&F University, Yangling, China

[a] Email: zanlinsen@163.com

Supplementary information 1 Fig S1

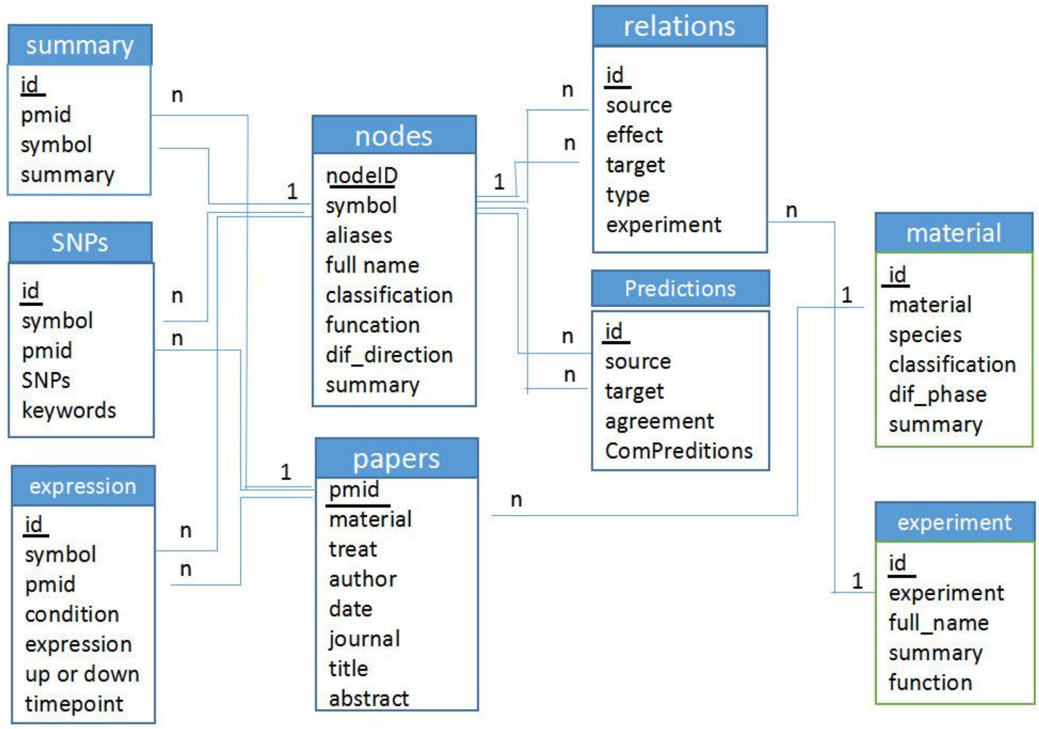

Fig S1. The database table structure

Supplementary 2-Handbook of ARN

Handbook of ARN database

http://210.27.80.93/arn/

Menu

1. Contents of ARN
2. Definitions
3. Examples

ARN

Contents of ARN Database

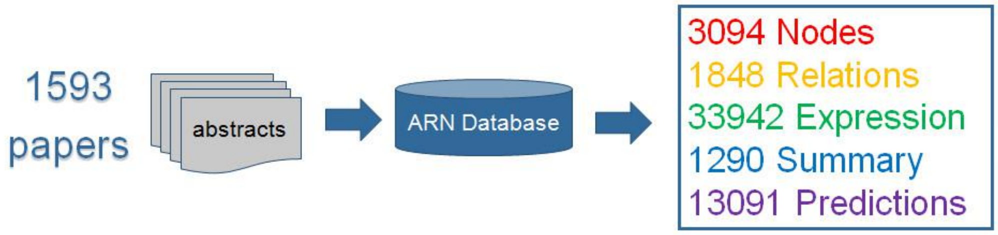

Paper= Abstract + Material + Methods + Datas ......

Definitions

1. **Node:** genes or miRs regulate adipogenic differentiation
2. **Symbol:** Official Symbol of nodes in NCBI-gene
3. **link:** Relationship between the nodes
4. **IF(%):** quantification the influence of node on adipogenesis
5. **Agreement:** quantification the reliability of predictions, 100 means verified

How to use ARN database

Example 1
basic search

Example 2
Node analysis

Example 3
Paper analysis

Example 4
problem oriented
Data analysis

Example 1 basic search

1

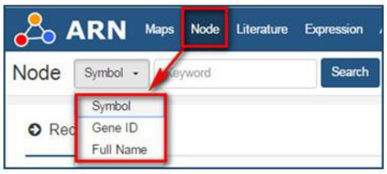

Node page
Users can search by node Symbol, Gene ID or full name

2

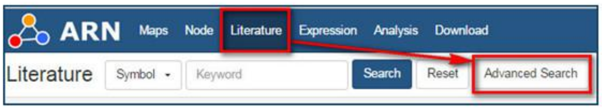

Literature page
The "advanced search" can screen articles according to the types of material, PMID or other details

Example 2 Node analysis (Sp1)

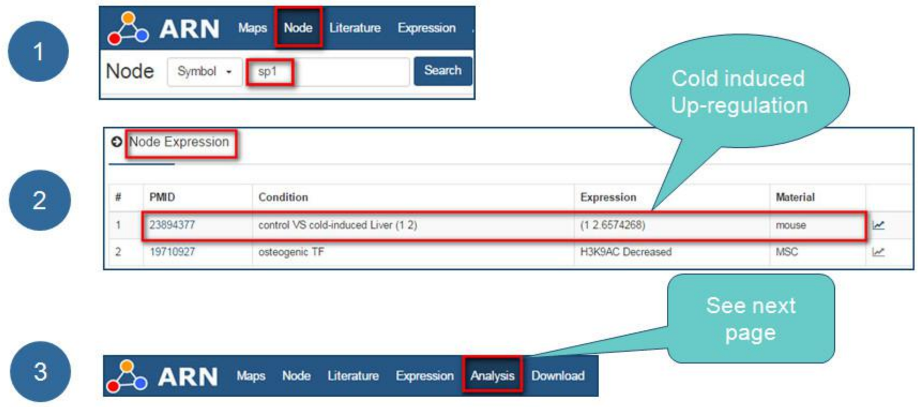

Example 2 Node analysis (Sp1)

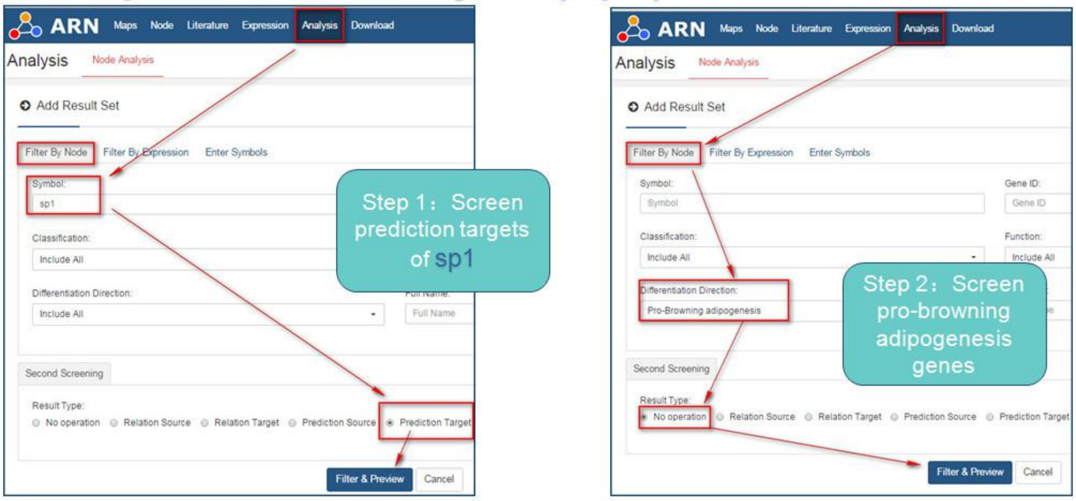

Example 2 Node analysis (Sp1)

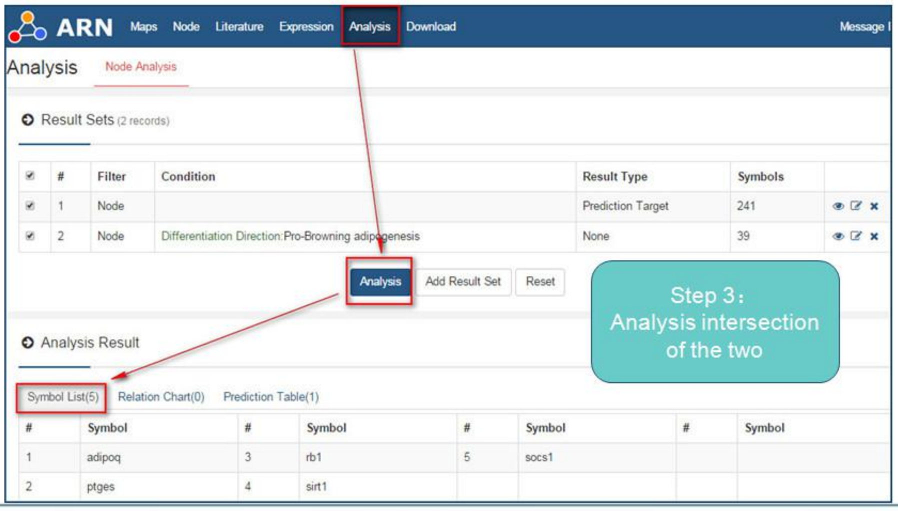

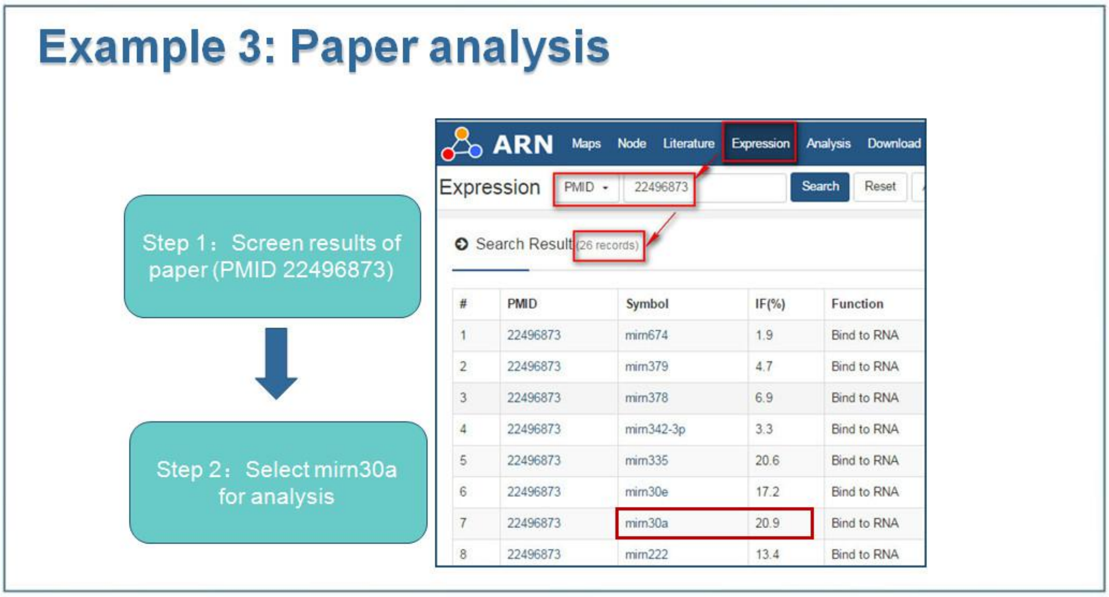

#	PMID	Symbol	IF(%)	Function
1	22496873	mirn674	1.9	Bind to RNA
2	22496873	mirn379	4.7	Bind to RNA
3	22496873	mirn378	6.9	Bind to RNA
4	22496873	mirn342-3p	3.3	Bind to RNA
5	22496873	mirn335	20.6	Bind to RNA
6	22496873	mirn30e	17.2	Bind to RNA
7	22496873	mirn30a	20.9	Bind to RNA
8	22496873	mirn222	13.4	Bind to RNA

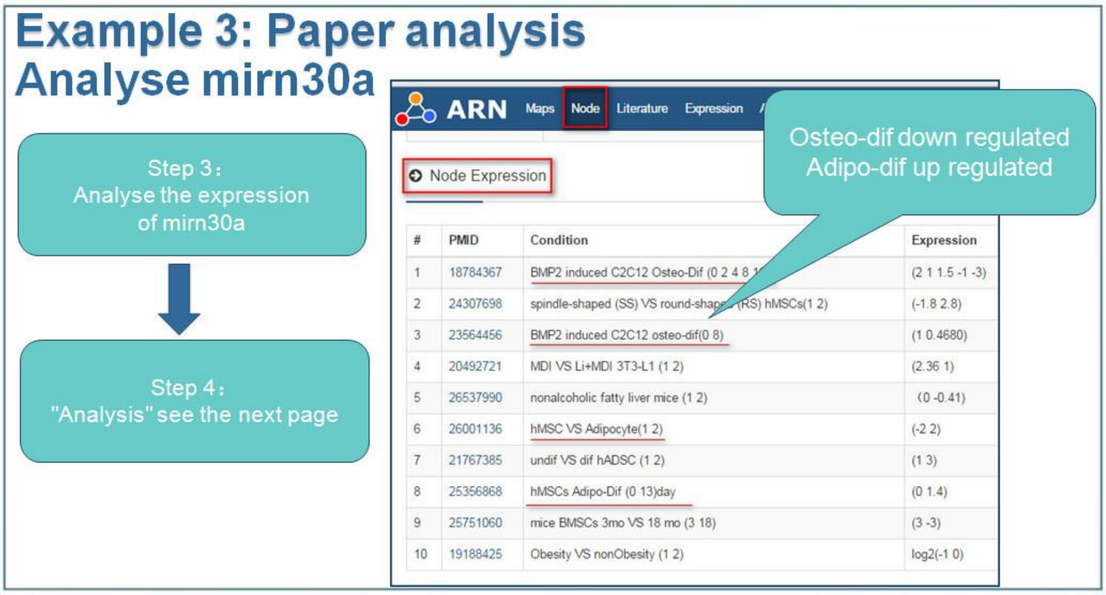

#	PMID	Condition	Expression
1	18784367	BMP2 induced C2C12 Osteo-Dif (0 2 4 8 [illegible]	(2 1 1.5 -1 -3)
2	24307698	spindle-shaped (SS) VS round-shap[illegible](RS) hMSCs(1 2)	(-1.8 2.8)
3	23564456	BMP2 induced C2C12 osteo-dif(0 8)	(1 0.4680)
4	20492721	MDI VS Li+MDI 3T3-L1 (1 2)	(2.36 1)
5	26537990	nonalcoholic fatty liver mice (1 2)	(0 -0.41)
6	26001136	hMSC VS Adipocyte(1 2)	(-2 2)
7	21767385	undif VS dif hADSC (1 2)	(1 3)
8	25356868	hMSCs Adipo-Dif (0 13)day	(0 1.4)
9	25751060	mice BMSCs 3mo VS 18 mo (3 18)	(3 -3)
10	19188425	Obesity VS nonObesity (1 2)	log2(-1 0)

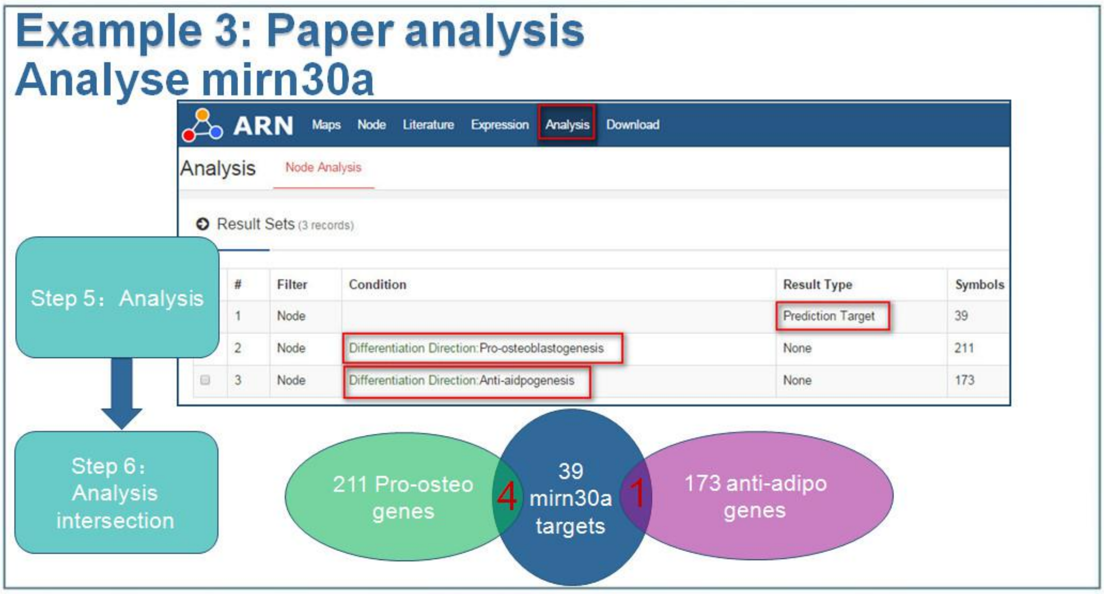

#	Filter	Condition	Result Type	Symbols
1	Node		Prediction Target	39
2	Node	Differentiation Direction:Pro-osteoblastogenesis	None	211
3	Node	Differentiation Direction:Anti-aidpogenesis	None	173

Example 3: Paper analysis
Analyse mirn30a

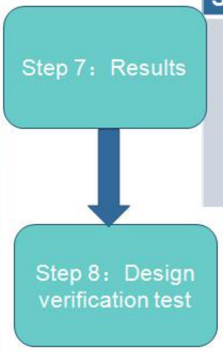

Source	target	IF(%)	分化方向
mirn30a	tsc22d3	12.3	Anti-adipogenesis
	cbfb	4.9	Pro-osteogenesis
	sox12	1.2	
	tsc22d3	12.3	
	wnt5a	5.3	

Example 4 problem oriented Data analysis

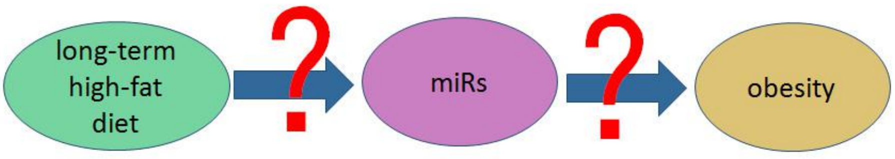

Is long-term high-fat diet promote adipogenesis by regulating miRs? How? ?

Example 4: problem oriented Data analysis

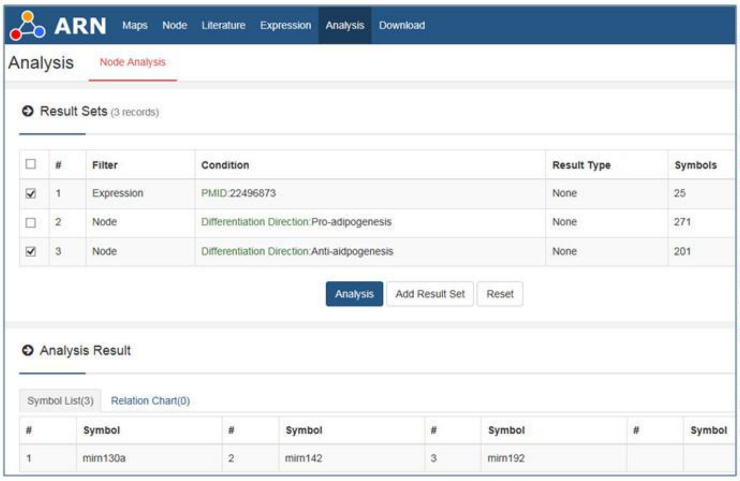

Step 1:
http://210.27.80.93/arn/Analysis/
==> Add Result Set
==> Filter By Expression
==> PMID: 22496873
==> Filter & Preview
==> Save Result Set
Step 2:
==> Add Result Set
==> Filter By Node
==> Differentiation Direction: Pro-adipogenesis
==> Filter & Preview
==> Save Result Set
Step 3:
==> Add Result Set
==> Filter By Node
==> Differentiation Direction: Anti-adipogenesis
==> Filter & Preview
==> Save Result Set

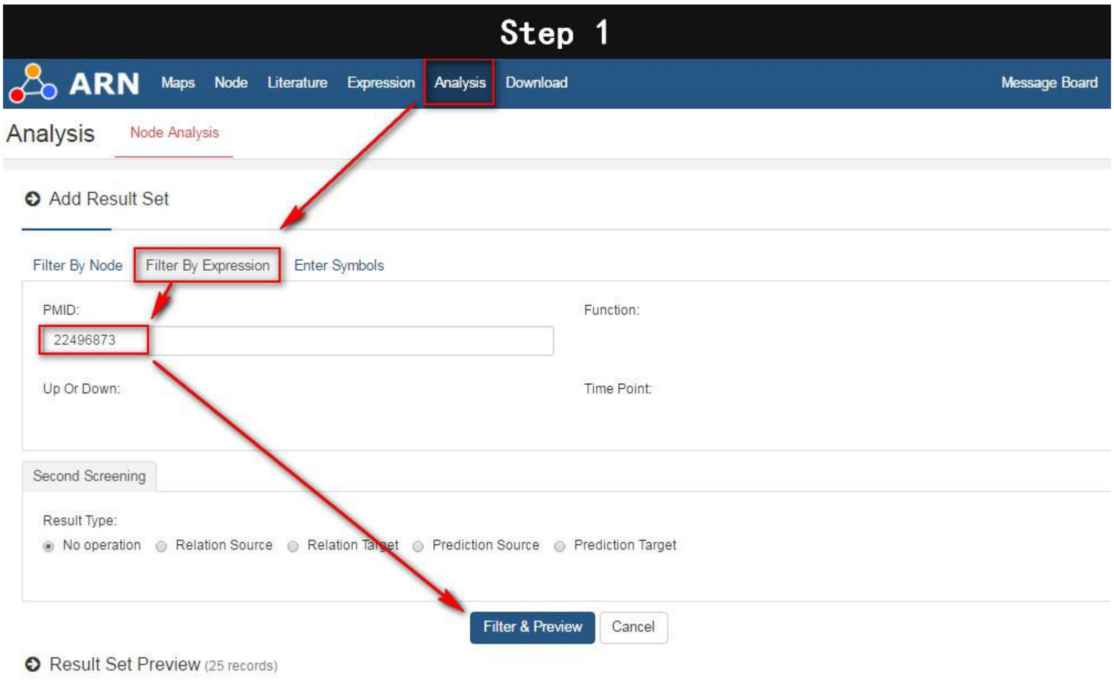
Step 1
ARN
Maps
Node
Literature
Expression
Analysis
Download
Message Board
Analysis
Node Analysis
Add Result Set
Filter By Node
Filter By Expression
Enter Symbols
PMID:
22496873
Function:
Up Or Down:
Time Point:
Second Screening
Result Type:
No operation
Relation Source
Relation Target
Prediction Source
Prediction Target
Filter & Preview
Cancel
Result Set Preview (25 records)

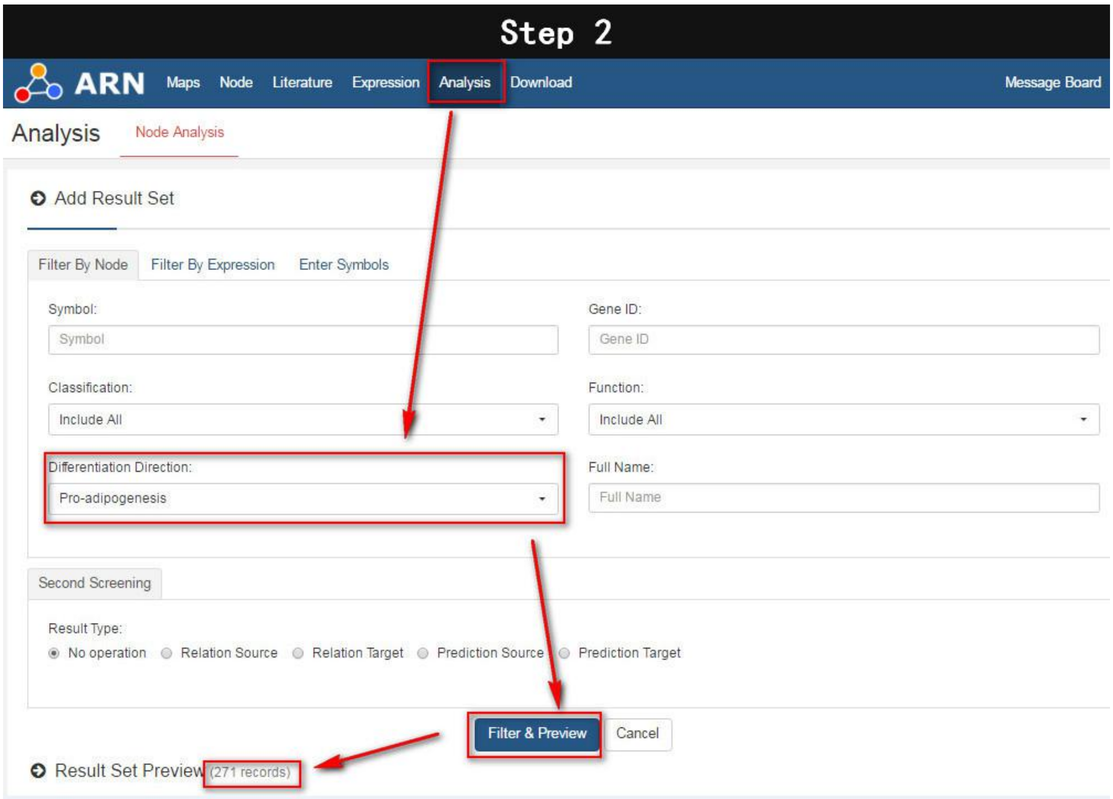
Step 2
ARN
Maps
Node
Literature
Expression
Analysis
Download
Message Board
Analysis
Node Analysis
Add Result Set
Filter By Node
Filter By Expression
Enter Symbols
Symbol:
Symbol
Gene ID:
Gene ID
Classification:
Include All
Function:
Include All
Differentiation Direction:
Pro-adipogenesis
Full Name:
Full Name
Second Screening
Result Type:
No operation
Relation Source
Relation Target
Prediction Source
Prediction Target
Filter & Preview
Cancel
Result Set Preview (271 records)

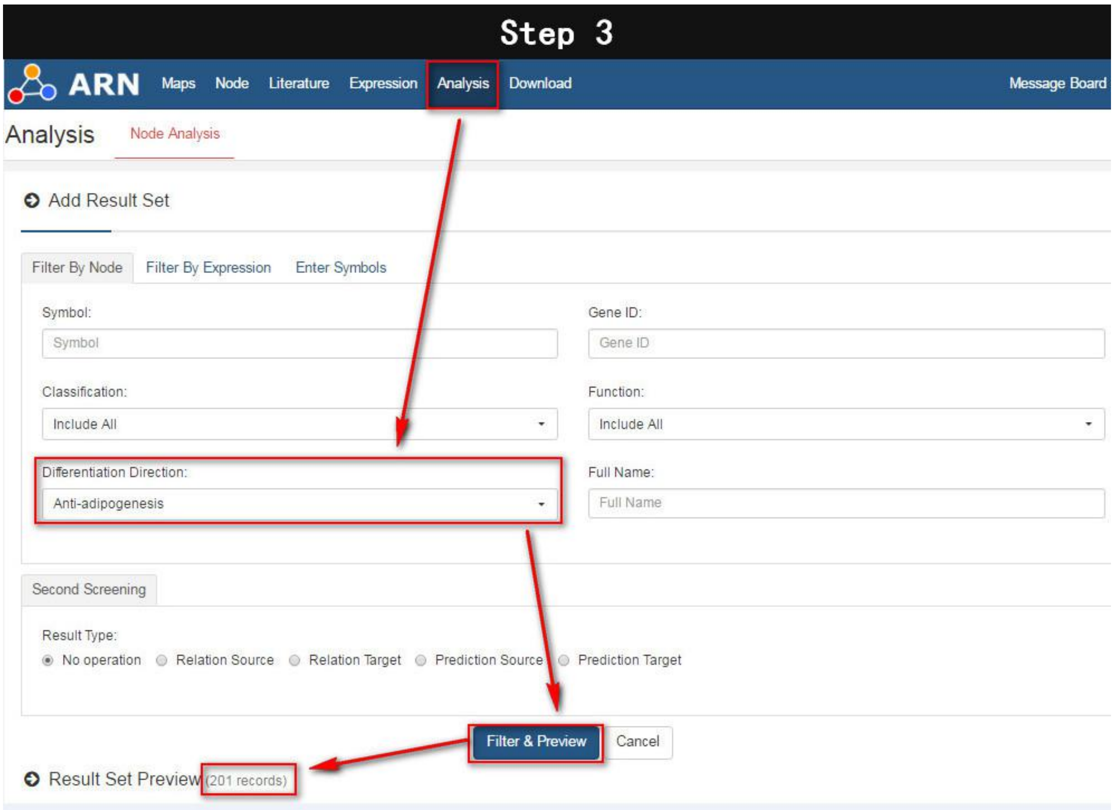
Step 3
ARN
Maps
Node
Literature
Expression
Analysis
Download
Message Board
Analysis
Node Analysis
Add Result Set
Filter By Node
Filter By Expression
Enter Symbols
Symbol:
Symbol
Gene ID:
Gene ID
Classification:
Include All
Function:
Include All
Differentiation Direction:
Anti-adipogenesis
Full Name:
Full Name
Second Screening
Result Type:
No operation
Relation Source
Relation Target
Prediction Source
Prediction Target
Filter & Preview
Cancel
Result Set Preview
(201 records)

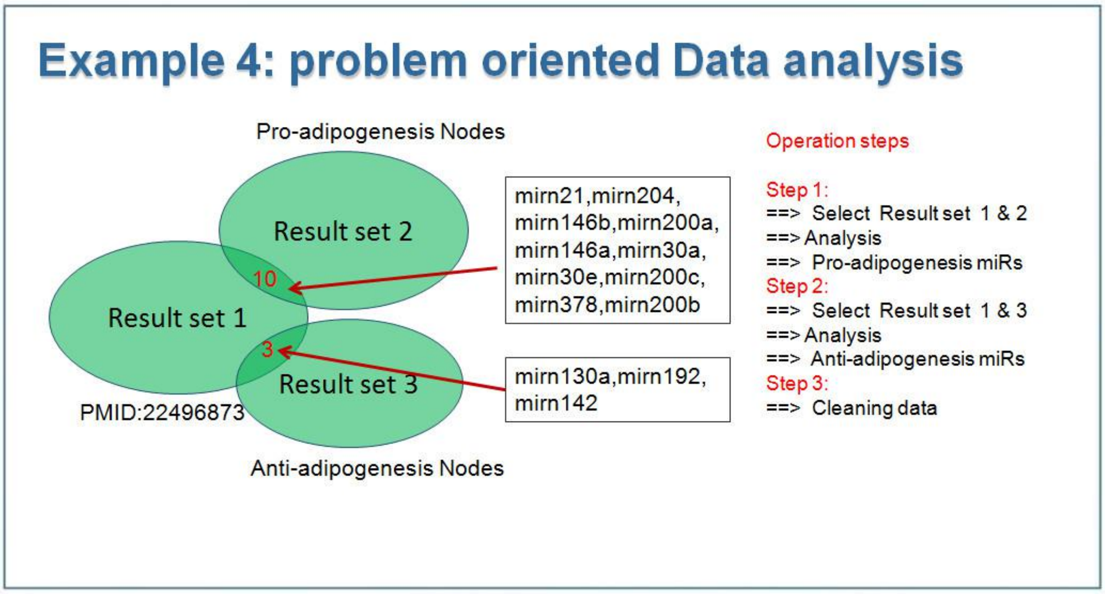
Example 4: problem oriented Data analysis
Pro-adipogenesis Nodes
Result set 2
Result set 1
Result set 3
10
3
PMID:22496873
Anti-adipogenesis Nodes
mirn21,mirn204,
mirn146b,mirn200a,
mirn146a,mirn30a,
mirn30e,mirn200c,
mirn378,mirn200b
mirn130a,mirn192,
mirn142
Operation steps
Step 1:
==> Select Result set 1 & 2
==> Analysis
==> Pro-adipogenesis miRs
Step 2:
==> Select Result set 1 & 3
==> Analysis
==> Anti-adipogenesis miRs
Step 3:
==> Cleaning data

Example 4: problem oriented Data analysis

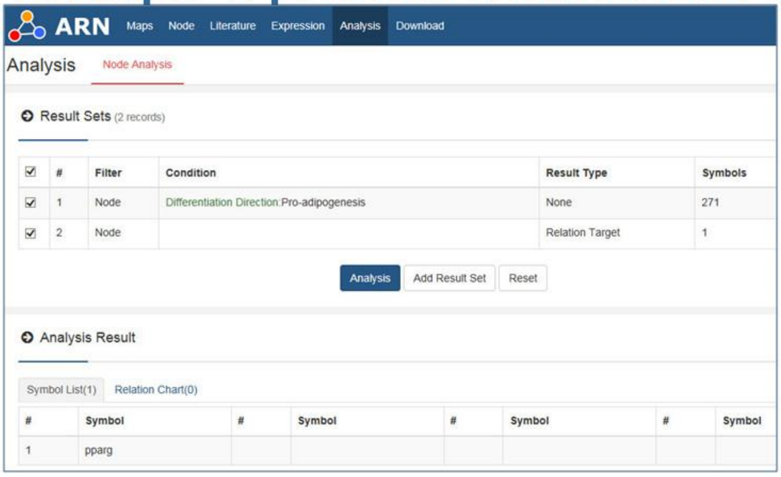

Step 1:
==> Add Result Set
==> Filter By Node
==> Differentiation Direction: Pro-adipogenesis
==> Filter & Preview
==> Save Result Set
Step 2:
==> Add Result Set
==> Filter By Node
==> Symbol: mirn130a
==> Second Screen: Relation Target
==> Filter & Preview
==> Save Result Set
Step 3:
==> Analysis
For the other 12 miRs, repeat the above steps.

Example 4: problem oriented Data analysis

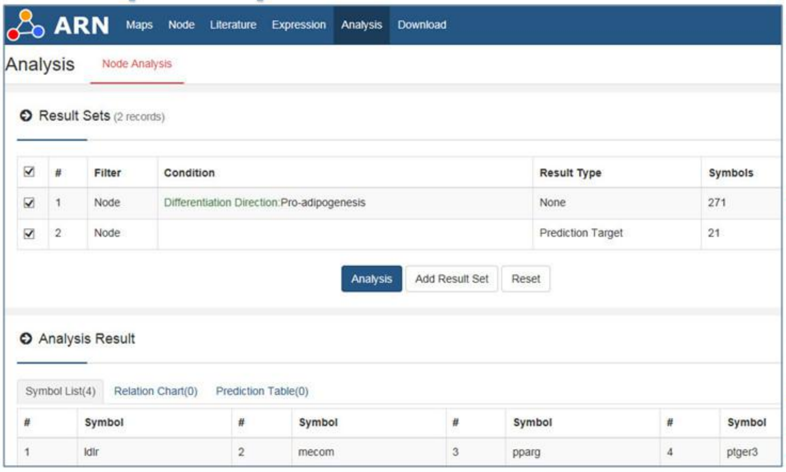

Step 1:
==> Add Result Set
==> Filter By Node
==> Differentiation Direction: Pro-adipogenesis
==> Filter & Preview
==> Save Result Set
Step 2:
==> Add Result Set
==> Filter By Node
==> Symbol: mirn130a
==> Second Screen: Prediction Target
==> Filter & Preview
==> Save Result Set
Step 3:
==> Analysis
For the other 12 miRs, repeat the above steps.

Example 4: problem oriented Data analysis

No.	Differentiate direction	Source	Category	Target
1	Pro-adipogenesis	mirn21	Prediction target	bach1
				nfat5
				reck
2		mirn204	Relation target	sirt1
				andcr
3		mirn146b	Relation target	brca1
				sirt1
			Prediction target	klf7
4		mirn200a	Prediction target	sirt1
				ctnnb1
				asxl1
				hdac4
				yap1
5		mirn146a	Relation target	kdm6b
				smad3
				wnt1
			Prediction target	brca1
6		mirn30a	Prediction target	tsc22d3
7		mirn30e	Prediction target	tsc22d3
				bach1
				nfat5
8		mirn200c	Prediction target	lepr
9		mirn378	Relation target	med13
10		mirn200b		None

No.	Differentiate direction	Source	Category	Target
11	Anti-aidpogenesis	mirn130a	Relation target	pparg
			Prediction target	ldlr
				ptger3
				mecom
12		mirn192	Relation target	scd
			Prediction target	rb1
				fndc3b
				ppp1cb
13		mirn142		None

Analysis Result
